# The association between parental depression and adolescent’s Internet addiction in South Korea

**DOI:** 10.1186/s12991-018-0187-1

**Published:** 2018-05-04

**Authors:** Dong-Woo Choi, Sung-Youn Chun, Sang Ah Lee, Kyu-Tae Han, Eun-Cheol Park

**Affiliations:** 1 Department of Public Health, Graduate School, Yonsei University, Seoul, Republic of Korea; 2 Institute of Health Services Research, Yonsei University, Seoul, Republic of Korea; 3 Department of Policy Research Affairs, National Health Insurance Service Ilsan Hospital, Koyang, Republic of Korea; 4 Department of Preventive Medicine, Yonsei University College of Medicine, 50 Yonsei-ro, Seodaemun-gu, Seoul, 03722 Republic of Korea

**Keywords:** Maternal depression, Internet addiction, Mental health, Adolescent, CESD-11, Internet Addiction Scale

## Abstract

**Background:**

A number of risk factors for Internet addiction among adolescents have been identified to be associated with their behavior, familial, and parental factors. However, few studies have focused on the relationship between parental mental health and Internet addiction among adolescents. Therefore, we investigated the association between parental mental health and children’s Internet addiction by controlling for several risk factors.

**Methods:**

This study used panel data collected by the Korea Welfare Panel Study in 2012 and 2015. We focused primarily on the association between Internet addiction which was assessed by the Internet Addiction Scale (IAS) and parental depression which was measured with the 11-item version of the Center for Epidemiologic Studies Depression Scale. To analyze the association between parental depression and log-transformed IAS, we conducted multiple regression analysis after adjusting for covariates.

**Results:**

Among 587 children, depressed mothers and fathers comprised 4.75 and 4.19%, respectively. The mean IAS score of the adolescents was 23.62 ± 4.38. Only maternal depression (β = 0.0960, *p* = 0.0033) showed higher IAS among children compared to nonmaternal depression. Strongly positive associations between parental depression and children’s Internet addiction were observed for high maternal education level, adolescents’ gender, and adolescent’s academic performance.

**Conclusions:**

Maternal depression is related to children’s Internet addiction; particularly, mothers who had graduated from the university level or above, male children, and children’s normal or better academic performance show the strongest relationship with children’s Internet addiction.

## Introduction

The Internet has become an integral part of our daily lives. We have come to depend so much on the Internet that we are unable to imagine a world without it. However, this dependence causes Internet addiction. Although Internet addiction is not yet recognized as an established disorder, it is considered an emerging behavioral problem, particularly among adolescents [1].

While more than 80% of the population in the United Kingdom, United States, and Asia have access to the Internet, a smaller proportion of the population in South America (45–55%) have Internet access, and the proportion of young Internet users in Africa and the Middle East has increased by about 3000% over the past decade [2]. According to international research, over 30% of children under the age of 2 used tablets or smartphones, while 80% of teenagers owned similar devices [3].

A number of risk factors for Internet addiction among adolescents have been identified to be associated with their behavior, familial, and parental factors such as family relationships, dysfunction families, parental attitudes, and parenting styles [4–8]. However, few studies have focused on the relationship between parental mental health and Internet addiction among adolescents [9]. Parental depression has extensive consequences on family life and children’s social adjustment and mental health, and these results induce major psychiatric problems such as depression and anxiety among these children [10, 11]. Therefore, parental mental health problems need to be managed for their own good as well as that of their children.

In this study, we focused on Internet addiction among adolescents in relation to parental depression, considering other risk factors. We hypothesized that parental mental health problems are associated with a negative impact on Internet addiction among children, and that particularly, the mother’s role is more important in curbing this addiction compared to the father.

## Methods

### Study population

This study used panel data collected by the Korea Welfare Panel Study (KOWEPS) between 2012 and 2015 from nationally representative samples of Korean households with two-stage-stratified cluster sampling. The KOWEPS included information of 18,856 participants from 7072 households, regarding socioeconomic status, health status, and insurance and welfare status. Moreover, it included an additional survey of students aged 11–19 years, regarding health status, education status, and school life triennially. Among 775 children who participated in this study, 188 provided no answer for survey questionnaires about variables including school type, school record, stress and academic performance, mental health score indexes, parental depression, and socioeconomic status, and were excluded. Accordingly, 587 children were included in this study.

### Variables

Internet addiction was assessed by the Internet Addiction Scale (IAS) for children, which was developed by the National Information Society Agency. This scale was a self-assessment tool developed as a short form of the *K*-scale considering it was too long for children to fill out and hardly reflected the characteristics appropriately in Korea [12]. It consisted of 20 questions for each of the four categories assessing Internet addiction from ‘never true’ to ‘always true.’ Therefore, the IAS score was obtained by summing the individual values of the answers to the 20 questions. This standard scale was validated to screen and evaluate Internet addiction by previous studies [13, 14].

We focused primarily on parental depression. Parental depression was measured with the 11-item version of the Center for Epidemiologic Studies Depression Scale (CESD-11). The CESD-11 is a shorter version of the 20-item CESD and is a self-reported screening tool which is well validated [15]. The CESD-11 consisted of 11 questions for each of the four categories measuring depression symptoms and the total score was calculated by adding the scores for all questions and multiplying this value by 20/11. Depression was diagnosed by obtaining a CESD-11 score of above 16.

Covariates were used such as sex, parental economic status (‘salary employee,’ ‘employer or self-employed,’ and ‘not employed or unemployed’), parental education level (‘high school or below’ and ‘university or above’), household income (‘low,’ ‘mid-low,’ ‘mid-high,’ ‘high’), school type (‘elementary school’ and ‘high school’), academic performance (‘poor or very poor,’ ‘normal,’ and ‘good or very good’), academic achievement stress and score on the Korean version of the Child Behavior Checklist (K-CBCL) [16, 17]. The Academic Achievement Stress Scale was measured by four items measuring students’ perceived stress from score 1 to 4 (performance, homework, concern of College entrance exams, and burn-out for study). Total score is the sum of each item that indicates a level of adolescent’s perceived stress. The K-CBCL consists of five items, including depression and anxiety, attention problems, social problems, delinquency, and aggression. Each question has The K-CBCL scale was the modified Korean version of Achenbach’s CBCL and has three answer categories ranging from ‘never felt,’ ‘sometimes felt,’ and ‘strongly felt,’ and each category score is valued as 0, 1, or 2, and the total score is obtained by summing the scores of all items.

### Statistical analysis

For all statistical analyses, we used weights to improve the representativeness of the samples provided by the KOWEPS. First, the frequencies and percentages of the study population and weighted mean of IAS score for each variable were determined by *t* test and analysis of variance. Thereafter, we performed a log-transformation of the IAS score to improve normality. To analyze the association between parental depression and log-transformed IAS, we performed multiple regression analysis after controlling for covariates such as parental economic status, parental education level, household income, adolescent’s sex, school type, academic performance, academic achievement stress score, and K-CBCL score. Finally, we performed subgroup analyses for the association between adolescents’ IAS score and different factors according to parental depression after adjusting for covariates. All statistical analyses were performed using SAS 9.4 (SAS Institute Inc., Cary, NC, USA), and *p*-values less than 0.05 were considered statistically significant.

## Results

Table 1 shows the general characteristics of the study population. The prevalence rates of maternal and paternal depression were 4.75 and 4.19%, respectively. Regarding education level, 56.53 and 43.47% of mothers and fathers had graduated from high school or below, respectively, and 43.47 and 51.19% of mothers and fathers had graduated from university or above, respectively. Of the children surveyed, 52.28% were males and 47.72% were females. The average IAS score for the adolescents was 23.62 ± 4.38.

**Table 1:** The general characteristics of the study population in this study

Variables	*N*/mean	% (weighted)/SD	IAS		
			Mean	SD	*p*-value
Parents					
Mother’s depression					0.0049
Yes	36	4.75	25.61	± 5.08	
No	551	95.26	23.53	± 4.33	
Father’s depression					0.9898
Yes	30	4.19	24.38	± 4.55	
No	557	95.81	23.59	± 4.38	
Mother’s economic status					0.0016
Salary employee	287	48.76	24.17	± 4.54	
Employer or self-employed	91	15.32	23.19	± 4.65	
Not employed or unemployed	209	35.92	23.08	± 3.99	
Father’s economic status					0.2665
Salary employee	400	71.19	23.75	± 4.66	
Employer or self-employed	157	24.32	23.20	± 3.55	
Not employed or unemployed	30	4.50	23.93	± 4.70	
Mother’s education level					0.1000
High school or under	347	56.53	23.90	± 4.62	
University or above	240	43.47	23.27	± 4.01	
Father’s education level					0.0003
High school or under	318	48.81	24.32	± 4.72	
University or above	269	51.19	22.97	± 3.86	
Household income					0.5061
Low	147	19.78	23.82	± 4.16	
Mid-low	149	22.97	23.37	± 4.08	
Mid-high	148	25.63	24.04	± 4.59	
High	143	31.62	23.36	± 4.72	
Adolescents					
Gender					< 0.0001
Male	293	52.28	24.44	± 4.62	
Female	294	47.72	22.73	± 3.98	
School type					
Elementary school	303	41.70	23.24	± 3.90	
High school	284	58.30	23.90	± 4.84	
Academic performance					< 0.0001
Bad or very bad	105	19.44	25.28	± 5.61	
Normal	199	36.50	23.70	± 4.72	
Good or very good	283	44.07	22.83	± 3.34	
Academic achievement stress score^a^	8.74	± 2.92			
Depression and anxiety score^a^	4.14	± 4.38			
Attention problems score^a^	3.45	± 3.79			
Social problem score^a^	2.35	± 2.84			
Delinquency score^a^	0.73	± 1.45			
Aggression score^a^	2.79	± 3.69			
Total	587	100.00	23.64	± 4.38	

^a^Mean and standard deviation (SD) of the continuous independent variables in this study

Table 2 shows the results of the multiple regression analysis performed to investigate the relationship between factors and the Internet addiction among adolescents. As shown in Table 2, maternal depression (β = 0.0960, *p* = 0.0033) yielded higher IAS compared to nonmaternal depression, unlike paternal depression which did not show significantly different findings between groups (*p* = 0.7555). Maternal education at the high school level or below (β = 0.0380, *p* = 0.0173) yielded higher IAS than those with education at the university level or above. On the other hand, paternal education level did not show statistically significant findings (*p* = 0.2132). Male children showed higher IAS (β = 0.0815, *p* < 0.0001) compared to that of female children. Moreover, children who performed poorly academically had higher IAS (β = 0.0480, *p* = 0.0286) compared to those with good or very good academic performance.

**Table 2:** The results of multiple regression analysis performed to investigate the relationship between factors and adolescent’s Internet addiction

Variables	Log-transformed IAS		
	β	SE	*p*-value
Parents			
Mother’s depression			
Yes	0.0960	0.0325	0.0033
No	Ref	–	–
Father’s depression			
Yes	− 0.0107	0.0343	0.7555
No	Ref	–	–
Mother’s economic status			
Salary employee	0.0296	0.0156	0.0575
Employer or self-employed	− 0.0090	0.0237	0.7054
Not employed or unemployed	Ref	–	–
Father’s economic status			
Salary employee	0.0041	0.0289	0.8865
Employer or self-employed	− 0.0244	0.0303	0.4210
Not employed or unemployed	Ref	–	–
Mother’s education level			
High school or under	0.0380	0.0159	0.0173
University or above	Ref	–	–
Father’s education level			
High school or under	− 0.0203	0.0162	0.2132
University or above	Ref	–	–
Household income			
Low	− 0.0033	0.0213	0.8777
Mid-low	− 0.0183	0.0190	0.3338
Mid-high	− 0.0173	0.0180	0.3368
High	Ref	–	–
Adolescents			
Gender			
Male	0.0815	0.0143	< 0.0001
Female	Ref	–	–
School type			
Elementary school	0.0178	0.0174	0.3046
High school	Ref	–	–
Academic performance			
Bad or very bad	0.0480	0.0219	0.0286
Normal	0.0175	0.0159	0.2715
Good or very good	Ref	–	–
Academic achievement stress score	0.0053	0.0028	0.0624
Depression and anxiety score	− 0.0002	0.0026	0.9341
Attention problems score	0.0088	0.0030	0.0029
Social problem score	0.0022	0.0041	0.5997
Delinquency score	− 0.0072	0.0077	0.3484
Aggression score	0.0127	0.0033	0.0001

**Fig. 1:**
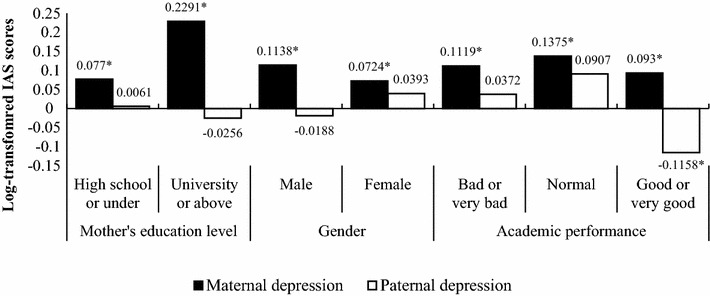
The results of subgroup analysis for the association between parental depression and log-transformed IAS according to different factors. *Statistically significant results (*p* < 0.05). ^§^Analyses were adjusted for the following covariates: economic status, father’s education level, household income, gender, school type, academic performance, academic achievement stress score, depression and anxiety score, attention problems score, social problem score, delinquency score, and aggression score

Figure 1 shows the results of the subgroup analysis for the association between parental depression and IAS according to the children’s maternal education level, adolescents’ gender, and academic performance. Adolescents’ mothers with depression who graduated from high school or below had higher IAS (β = 0.0770, *p* = 0.0187) compared to those who did not have depression. Likewise, among adolescents’ mothers who graduated from university or above, children with maternal depression (β = 0.2291, *p* < 0.0001) had higher IAS compared to those whose mothers did not have depression. However, there were not significant results in paternal depression (*p* > 0.05). Male children whose mothers had depression showed higher IAS (β = 0.1138, *p* = 0.0383) than those who had no depression. Female adolescents whose mothers had depression also had higher IAS (β = 0.0724, *p* = 0.0300) compared to those whose mothers had no depression. However, there was no statistically significant association for children of fathers with depression (male: p = 0.6209, female: *p* = 0.4951).

## Discussion

The association between parental depression and adolescent Internet addiction was the main finding of this study. As shown above, the most important finding was that maternal depression could result in Internet addiction among children. On the other hand, paternal depression was not significantly associated with the development of Internet addiction among children. Moreover, this result was also consistently maintained in the subgroup analysis according to high maternal education levels such as university or above, male children, and normal or better academic performance.

Previous studies have found an association between Internet addiction among children and family factors such as adolescent–parent conflict, parental monitoring, and parental attitude [4, 5]. Particularly, parental depression has been known as one of the major risk factors for adverse behavioral and emotional functioning among children by numerous studies and reviews [18–20]. Unfortunately, we could find just a few studies about the association between parental depression and Internet addiction among children. Lam found that the association between parental depression and Internet addiction among adolescents might reflect the relationship of the children’s depression status and their parents’ depression [9]. In addition, parental mental health also plays a significant role in the development of Internet addiction among children. Interestingly, our study found that only maternal depression adversely was associated with Internet addiction among children. It could be guessed that depression might adversely affect mothers’ parenting attitudes [21, 22]. South Koreans have a strong tendency of mother’s parenting responsibility [23]. Mothers take care of all for their children such as their schedule of school, private academy, and a college entrance examination [24]. In other words, children are more affected by mother’s care for children than father’s. Moreover, the association between maternal depression and parenting behavior was manifest most strongly for negative effect including increased hostility, high rates of negative interactions, being fewer responsive to child behavior, to communicate less effectively and to have fewer interactions with their children [25]. Therefore, mothers with depression could take less care of their children’s behaviors and mental development, and the quality of the relationship between this mother and her children might be worse compared to others who are not depressed [20, 26]. As a result, these factors might strongly be associated with the development of Internet addiction among children.

In particular, this finding showed a stronger correlation when the mother’s educational level was at the university level or above. The results of our study were contradictory to those of other studies. Lam’s study indicated that mother’s education level was not associated with Internet addiction among children, and another study also showed that parental educational attainment was only associated with male children’s addictive Internet use [9]. However, our study showed a correlation between maternal high education level and Internet addiction among children. Moreover, children’s Internet addiction showed a strong correlation with mother’s high education level when their mother had depression. Previous study found that mothers who had high education level provide better home environment for their children by educating musical instruments, special lessons, and books [27]. When high educated mother got depressed, it would be difficult to support their children compared to those who did not get depressed. Thus, we could guess that high educated mother was more strongly associated with the development of children’s Internet addiction.

Male adolescents had high potential Internet addiction compared to female adolescents. This finding is supported by the difference in Internet addiction between genders found in several studies, and male gender was one of the risk factors identified by Young and Greenfield [28–30]. They demonstrated that male adolescents tended to use the Internet mainly for purposes related to entertainment and leisure, whereas women use it primarily for interpersonal communication and educational assistance.

Below normal academic performance groups showed strong associations between mother’s depression and Internet addiction of their children. A previous study showed that poor academic performance was a risk factor for Internet addiction [31]. Moreover, children who had poor performance were likely to have strong mother’s care, because their mother would be worried about their children’s future [32]. Therefore, when their mother was depressed it might happen that children with below normal academic performance had a high potential Internet addiction because there was no mother’s care.

Although many studies and literature have focused on the association between mental health of children and maternal depression, it has not yet been clearly discovered that maternal mental health is directly associated with Internet addiction among their children. Therefore, this study may suggest that Internet addiction among children may not only be affected by their behavior and mental problems, but may also be affected by the mental health of the mother.

This study has several limitations that should be considered when interpreting our results. First, this study is a cross-sectional study; thus, caution should be exercised in interpreting causality between parental depression and children’s Internet addiction. Second, there are not enough previous studies which could support to the results of the relationship between parental depression and children’s Internet addiction. However, it is likely to be one of evidence for a possibility of the relationship between parental mental health and children’s Internet addiction. Third, there is a risk of recall bias because the data were collected via self-reported surveys and interviews to assess parental depression, children’s Internet addiction, and children’s mental health. Finally, although we included several lifestyle covariates as potential confounders, it is likely that we have not included all independent variables related to children’s Internet addiction.

Despite these limitations, this study has several strengths. First, we used data from a nationwide survey with randomly sampled data, increasing the representativeness of the data for the general Korean population. Compared with previous studies, this study focuses on the parental depression. Therefore, it suggests that children’s Internet addiction is not only association with their behaviors and mental health, but also by parental mental health. Moreover, it might serve as a new perspective on the causes of Internet addiction among children.

In conclusion, maternal depression is related to children’s Internet addiction; particularly, mothers who graduated from the university or higher, male children, and normal or better academic performance show the strongest relationship with children’s Internet addiction. To prevent children’s Internet addiction, parents’ mental health, as well as children’s behavior, needs to be managed. Especially, maternal care might be one of the important roles for alleviating their children’s Internet addiction. Therefore, mothers who have children need to manage their children to use Internet appropriately. The results of this study could provide other researchers with additional inspiration to study the impact of parental mental health and children’s Internet addiction. In addition, since there is a lack of research on parental depression and children’s Internet addiction, we hope that further research will be uncovered to clarify the association between them.

## References

[1] Young KS (1998). Internet addiction: the emergence of a new clinical disorder. Cyberpsychol Behav.

[2] World Internet Users Statistics and World Population Stats. http://www.internetworldstats.com/stats.htm. Accessed 11 Nov 2017.

[3] Health Online. 2013. http://www.pewinternet.org/files/old-media//Files/Reports/PIP_HealthOnline.pdf. Accessed 11 Nov 2017.

[4] Park SK, Kim JY, Cho CB (2008). Prevalence of internet addiction and correlations with family factors among South Korean adolescents. Adolescence.

[5] Yen J-Y, Yen C-F, Chen C-C, Chen S-H, Ko C-H (2007). Family factors of internet addiction and substance use experience in Taiwanese adolescents. CyberPsychol Behav.

[6] Li W, Garland EL, Howard MO (2014). Family factors in internet addiction among Chinese youth: a review of English- and Chinese-language studies. Comput Hum Behav.

[7] Ko CH, Wang PW, Liu TL, Yen CF, Chen CS, Yen JY (2015). Bidirectional associations between family factors and internet addiction among adolescents in a prospective investigation. Psychiatry Clin Neurosci.

[8] Xiuqin H, Huimin Z, Mengchen L, Jinan W, Ying Z, Ran T (2010). Mental health, personality, and parental rearing styles of adolescents with internet addiction disorder. Cyberpsychol Behav Soc Netw.

[9] Lam LT (2015). Parental mental health and internet addiction in adolescents. Addict Behav.

[10] Lieb R, Isensee B, Höfler M, Pfister H, Wittchen H (2002). Parental major depression and the risk of depression and other mental disorders in offspring: a prospective-longitudinal community study. Arch Gen Psychiatry.

[11] Nomura Y, Wickramaratne PJ, Warner V, Mufson L, Weissman MM (2002). Family discord, parental depression, and psychopathology in offspring: ten-year follow-up. J Am Acad Child Adolesc Psychiatry.

[12] Kim DI, Chung YJ, Lee EA, Kim DM, Cho YM (2008). Development of internet addiction proneness scale-short form (KS scale). Korean Counsel Assoc.

[13] Jang KW, Lee JH (2007). Development and validation of the Korean version of the game addiction/engagement scale (KGAES). Korean J Health Psychol.

[14] Kang M, Oh I (2002). Development of internet addiction scale for Korean adolescent. Korean J Educ Psychol.

[15] Kohout FJ, Berkman LF, Evans DA, Cornoni-Huntley J (1993). Two shorter forms of the CES-D (Center for Epidemiological Studies Depression) depression symptoms index. J Aging Health.

[16] Oh K, Lee H, Hong K, Ha E (1997). K-CBCL.

[17] Achenbach TM, Edelbrock C (1991). Manual for the child behavior checklist/4–18 and 1991 profile.

[18] Downey G, Coyne JC (1990). Children of depressed parents: an integrative review. Psychol Bull.

[19] Goodman SH, Gotlib IH (2002). Children of depressed parents: mechanisms of risk and implications for treatment.

[20] Olsson MB, Hwang C (2001). Depression in mothers and fathers of children with intellectual disability. J Intellect Disabil Res.

[21] Cox JE, Buman M, Valenzuela J, Joseph NP, Mitchell A, Woods ER (2008). Depression, parenting attributes, and social support among adolescent mothers attending a teen tot program. J Pediatr Adolesc Gynecol.

[22] Hoffman C, Crnic KA, Baker JK (2006). Maternal depression and parenting: implications for children’s emergent emotion regulation and behavioral functioning. Parenting.

[23] Jahng KE, Lim HJ (2015). A study on the predictors of parenting responsibility of mothers with infants. Korean J Early Childhood Educ.

[24] Kim H-O, Hoppe-Graff S (2001). Mothers roles in traditional and modern korean families: the consequences for parental practices and adolescent socialization. Asia Pac Educ Rev.

[25] Lovejoy MC, Graczyk PA, O’Hare E, Neuman G (2000). Maternal depression and parenting behavior: a meta-analytic review. Clin Psychol Rev.

[26] Burke L (2003). The impact of maternal depression on familial relationships. Int Rev Psychiatry.

[27] Carneiro P, Meghir C, Parey M (2013). Maternal education, home environments, and the development of children and adolescents. J Eur Econ Assoc.

[28] Ko C-H, Yen J-Y, Yen C-F, Chen C-S, Wang S-Y (2008). The association between internet addiction and belief of frustration intolerance: the gender difference. Cyberpsychol Behav.

[29] Greenfield DN (1999). Psychological characteristics of compulsive internet use: a preliminary analysis. Cyberpsychol Behav.

[30] Bakken IJ, Wenzel HG, Götestam KG, Johansson A, Øren A (2009). Internet addiction among Norwegian adults: a stratified probability sample study. Scand J Psychol.

[31] Chen Y-L, Chen S-H, Gau SS-F (2015). ADHD and autistic traits, family function, parenting style, and social adjustment for internet addiction among children and adolescents in Taiwan: a longitudinal study. Res Dev Disabil.

[32] Baker DP, Stevenson DL (1986). Mothers’ strategies for children’s school achievement: managing the transition to high school. Sociol Educ.

